# Exercise Craving: A Scale Development Study

**DOI:** 10.1002/hsr2.72225

**Published:** 2026-03-30

**Authors:** İsmail İlbak, Mehmet Akarsu, Ahmet Yasuntimur, Serkan Düz, Gabriela Kavalirova, Ladislav Cepicka

**Affiliations:** ^1^ Institute of Health Sciences İnönü University Malatya Türkiye; ^2^ Department of Physical Education and Sport İnönü University Malatya Türkiye; ^3^ Social Sciences Institute İnönü University Malatya Türkiye; ^4^ Department of Coaching Education, Faculty of Sport Sciences İnönü University Malatya Türkiye; ^5^ Department of Physical Education and Sport University of West Bohemia Czech Republic

**Keywords:** behavioral craving, deprivation, exercise addiction, exercise craving

## Abstract

**Background and Aim:**

Exercise craving refers to an uncontrollable urge to engage in exercise, accompanied by cognitive and emotional responses when deprived of it. Although several instruments exist to measure exercise addiction, there is no valid and reliable tool specifically designed to assess exercise craving. Addressing this gap is essential to better understand the psychological and behavioral outcomes of exercise deprivation. This study aimed to develop a valid and reliable scale to evaluate exercise craving levels in individuals unable to exercise.

**Methods:**

The research was conducted with a survey model in five stages. First, a comprehensive literature review and expert evaluations were used to generate an item pool. The preliminary version was piloted with 30 participants and revised. The finalized version was then applied to a sample of 222 volunteers. Exploratory Factor Analysis (EFA) and Confirmatory Factor Analysis (CFA) were performed to examine validity. Reliability was assessed using test‐retest, Cronbach's alpha, composite reliability (CR), and average variance extracted (AVE).

**Results:**

EFA revealed a two‐factor structure, and one item was removed due to low loading, resulting in an eight‐item scale that explained 74.628% of the variance. The first factor, exercise obsession, captured cognitive and emotional reactions to deprivation, while the second factor, effects of deprivation, reflected its negative influence on mood and daily functioning. CFA results confirmed a good model fit (*χ*²/df = 2.327, RMSEA = 0.077, SRMR = 0.030, CFI = 0.995, TLI = 0.990). Reliability analyses showed strong internal consistency (Cronbach's alpha = 0.897).

**Conclusion:**

The exercise craving scale is proposed as a valid and reliable tool for assessing the psychological and cognitive consequences of exercise deprivation.

## 1 Introduction

Exercise plays a crucial role in enhancing both physical and mental health, making it one of the fundamental determinants of quality of life [1, 2]. Regular exercise strengthens the cardiovascular system [3, 4, 5], reduces stress levels [6, 7, 8], and contributes significantly to individual and societal well‐being. Nevertheless, when exercise is performed excessively or uncontrollably, its benefits may turn into adverse outcomes such as overtraining syndrome [9] or exercise addiction [10].

Exercise addiction is defined as the persistent engagement in excessive exercise despite physical, psychological, or social harm, and is often accompanied by an inability to reduce or stop exercising [11]. Regular exercisers may also be forced to interrupt their routines due to workload, injuries, motivational decline, pandemic restrictions, or natural disasters [12]. In such situations, individuals may experience a strong urge or craving to exercise, reflecting an intense psychological state of desire independent of addiction. This indicates that exercise craving can occur not only among individuals with exercise addiction but also among those with regular, non‐pathological exercise habits.

Although the concept of craving has traditionally been associated with substance use, it has also been explored in behavioral contexts [13]. Scholars have investigated its definitions and applications across domains [14, 15, 16, 17]. Within this framework, exercise craving refers to the intense longing for exercise during periods when exercising is not possible [12]. Ilbak et al. [12] identified two types of exercise craving: (1) craving rooted in addiction, where exercise functions as a compulsive behavior, and (2) craving arising from uncontrollable circumstances, where individuals fear deterioration in their physical condition.

It is important to conceptually differentiate exercise craving from related constructs such as exercise addiction and exercise motivation. While exercise addiction reflects a maladaptive pattern of behavior with harmful consequences, and exercise motivation concerns the reasons driving participation in exercise [18], exercise craving specifically captures the acute and affectively charged desire to engage in exercise [12]. Existing scales measure exercise addiction (e.g., Exercise Addiction Inventory) [19] or exercise motivation (e.g., Behavioral Regulation in Exercise Questionnaire) [20], but to date, no validated tool has been developed to assess exercise craving. Thus, the proposed Exercise Craving Scale (ECS) is intended to address this conceptual gap by focusing on the unique psychological experience of craving for exercise.

Qualitative research has provided valuable insights into this phenomenon, yet such methods have limitations, including researcher bias, difficulties in generalization, and challenges in ensuring reliability and validity [21, 22, 23]. To overcome these issues and enable systematic investigation, the development of a standardized measurement tool is necessary. The proposed scale is not only relevant for identifying craving in individuals with exercise addiction but also for assessing this experience among regular exercisers and the broader population.

Therefore, the present study aims to develop and validate a scale to measure exercise craving. By doing so, this study contributes to clarifying the scope of exercise craving, distinguishing it from related constructs, and providing researchers and practitioners with a reliable tool to examine its individual, social, and cultural dimensions. Ultimately, the scale may help in promoting healthy exercise behaviors while informing strategies to prevent maladaptive patterns.

## 2 Methodology

### 2.1 Research Design

This study was designed using a survey model, and the scale development process was conducted in five stages. In the first stage, a comprehensive literature review was performed to generate an item pool suitable for measuring the target construct, followed by a content evaluation of the items based on the opinions of six field experts. In the second stage, the preliminary version of the scale was administered to a pilot group of 30 participants, and necessary revisions were made based on the feedback obtained. In the third stage, the finalized version of the scale was applied to a sample of 222 individuals who voluntarily agreed to participate in the study. In the fourth stage, exploratory factor analysis (EFA) and confirmatory factor analysis (CFA) were conducted to test the structural validity of the scale. Reliability analyses were performed using the test‐retest method, Cronbach's alpha coefficient, as well as average variance extracted (AVE) and composite reliability (CR) values. In the fifth and final stage, the scale items were reviewed based on the analysis results, the scoring system was clarified, and the measurement instrument was finalized. This study adhered to publication ethics as approved by the “İnönü University Social and Human Sciences Research and Publication Ethics Committee” (date: 13‐11‐2024; session number: 18; approval number: 17).

### 2.2 Designing the Scale and Creating the Draft Form

As a result of the literature review, relevant studies were examined [12, 24, 25], and based on these findings, an item pool was created. The item pool consisted of 9 items, which were evaluated using a five‐point Likert‐type scale. Participants responded to the scale items as follows: “1 = strongly disagree, 2 = disagree, 3 = neutral, 4 = agree, 5 = strongly agree.”

### 2.3 Expert Review and Content Validity

In this study, the content validity ratio (CVR) was used during the scale development process. CVR is a method that relies on expert opinions to convert qualitative data into quantitative data, particularly in cases where pilot studies are not feasible [26]. The scale items were evaluated by six experts specializing in exercise, physical activity, and sports psychology, as well as experts knowledgeable in measurement and evaluation. Based on the experts' feedback, the CVR was calculated.

During the calculation of the CVR, expert opinions were collected for each item, and items with a CVR greater than zero were retained if more than half of the experts deemed them appropriate. Initially, items with negative or zero CVR values were excluded, and then items with positive CVR values were analyzed. According to the minimum thresholds determined by Veneziano and Hooper [27], a minimum CVR value of 0.99 was required when consulting six experts.

The content validity index (CVI) was obtained from the average CVI of the items that met the significance level of *α* = 0.05 and were intended to be included in the final scale form [28]. Based on expert evaluations, items 3, 5, and 9 were revised. After these revisions, the CVI was recalculated and found to be 1.0. Since the obtained CVI value exceeded 0.99, the content validity of the scale was deemed high. This finding highlights the reliability of the measurement tool in accurately assessing exercise cravings [29].

### 2.4 Pilot Study

A pilot study was conducted to evaluate the clarity, comprehensibility, and technical functionality of the draft version of the ECS. A heterogeneous group of 30 participants, aged between 18 and 55, was selected to represent individuals with varying exercise habits and tendencies. The main objective was to assess whether the ECS items were easily understandable, clearly worded, and feasible for use within the target population. Based on the feedback obtained during this phase, minor linguistic and structural modifications were made to enhance clarity and ease of comprehension. Following these refinements, the draft version of the ECS was finalized with 9 items. This pilot study also served to confirm the applicability of the administration process and provided a methodological foundation for subsequent analyses of the scale's reliability and validity.

### 2.5 Participants

The minimum required sample size for this study was calculated using G*Power software (version 3.1.9.7; University of Düsseldorf, Düsseldorf, Germany). An F‐test was selected as the test type, with “linear multiple regression: fixed model, *R*² deviation from zero” chosen as the statistical test and “a priori: compute required sample size—given *α*, power, and effect size” as the analysis type. The effect size was set at 0.15 (moderate effect), the *α* error probability (significance level) at 0.05, the statistical power (1 − β error probability) at 0.95, and the number of predictors (items/factors) at 9. The analysis indicated that a minimum of 166 participants was required.

To exceed this threshold and ensure robust statistical power, a total of 222 participants were ultimately recruited. Participants were recruited through a convenience sampling strategy. Recruitment announcements were made in local fitness centers, university sports facilities, and online platforms (e.g., social media groups and mailing lists) to reach individuals with varying exercise habits. Participation was voluntary, and individuals were informed of the study's purpose and procedures prior to enrollment. Written informed consent was obtained from all participants, and ethical principles were observed throughout the data collection process.

### 2.6 Inclusion and Exclusion Criteria

The inclusion criteria for participation in the study were as follows: engaging in exercise at least once a week for a minimum of 30 min, voluntarily agreeing to participate, signing a written informed consent form, being mentally healthy, and having the cognitive ability to comprehend the study requirements. The exclusion criteria included individuals who had adopted an exercise habit within the past 6 months, those who were pregnant, individuals with alcohol or substance addiction, and those with language barriers that prevented them from understanding the survey questions. The descriptive statistics of the participant group are presented in Table 1.

**Table 1:** Characteristics of the participant group.

**Variable**		***F***	**%**
Gender	Male	136	61.261
	Female	86	38.739
Age	≤ 18 years	16	7.207
	19–25 years	86	38.739
	26–30 years	61	27.477
	31–35 years	25	11.261
	36–40 years	16	7.207
	≥ 41 years	18	8.108
Exercise experience (years)	≤ 1 year	56	25.225
	2–4 years	39	17.568
	5–7 years	53	23.874
	8–10 years	31	13.964
	≥ 11 years	43	19.369
Weekly exercise frequency (days)	1–2 days	91	40.991
	3–4 days	86	38.739
	5–6 days	32	14.414
	7 days	13	5.856

An examination of Table 1 reveals that the participant group consists of 222 individuals, of whom 61.3% are male and 38.7% are female. In terms of age distribution, the majority of participants fall within the 19–25 age group (38.7%), followed by those aged 26–30 (27.5%). The remaining age groups represent smaller proportions, with participants aged 41 and over comprising 8.1% of the total sample.

Regarding exercise experience, 25.2% of participants reported engaging in exercise for 1 year or less, 23.9% indicated having 5–7 years of experience, and 19.4% reported exercising for 11 years or more. In terms of weekly exercise frequency, the majority of participants engaged in exercise either 1–2 days (41.0%) or 3–4 days (38.7%) per week, while fewer participants reported exercising 5–6 days (14.4%) or every day (5.9%) per week. These findings indicate that the study sample consists of a diverse group of individuals with varying levels of exercise experience and frequency.

### 2.7 Statistical Analysis

The statistical procedures for this study were carried out using IBM SPSS Statistics version 25 (Armonk, NY, USA) and IBM AMOS version 24 (Chicago, IL, USA). Before performing the main analyses, the data set was screened for missing values, outliers, and compliance with normality assumptions. To explore the factor structure of the scale, an EFA was applied to the data set from the first sample group. Principal component analysis with varimax rotation was used as the extraction method. The adequacy of the data for factor analysis was assessed using the Kaiser–Meyer–Olkin (KMO) statistic and Bartlett's test of sphericity. The structure identified through EFA was further evaluated with a CFA conducted on data obtained from the second study group. CFA was performed with AMOS 24, and model fit was examined using several indices: root mean square error of approximation (RMSEA), goodness of fit index (GFI), adjusted goodness of fit index (AGFI), normed fit index (NFI), comparative fit index (CFI), incremental fit index (IFI), and root mean square residual (RMR). Following the confirmation of the factor structure, internal consistency was evaluated through Cronbach's alpha reliability coefficients for the subdimensions. Convergent validity was also assessed using CR and AVE.

## 3 Results

### 3.1 Exploratory Factor Analysis (EFA) and Confirmatory Factor Analysis (CFA)

To assess the suitability of the data for EFA, the Kaiser–Meyer–Olkin (KMO) test was conducted, yielding a value of 0.874, while Bartlett's sphericity test resulted in a value of 1498.071 (*p* < 0.01). Additionally, the internal consistency coefficient (Cronbach's alpha) was calculated as 0.897. These findings confirm that the data set is appropriate for conducting EFA [30].

During the item analysis, item 9 was found to have similar factor loadings across two dimensions, leading to its removal (Table 3). The remaining eight items were grouped under two factors. These two factors together explained 74.628% of the total variance. Consequently, the EFA results, evaluated through various statistical tests, indicate that the scale meets the criteria established for multifactorial scales and explains a significant portion of the variance. The explained total variance is presented in Table 2.

**Table 2:** Explained total variance.

Factor	Eigen value			Rotation sums of square loadings		
	Total	Variance %	Cumulative %	Total	Variance %	Cumulative %
1	4701	58,764	58,764	3057	38,216	38,216
2	1269	15,864	74,628	2913	36,412	74,628

**Figure 1:**
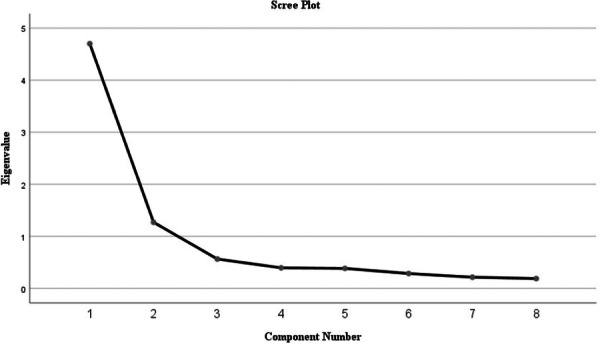
Scree plot.

When examining Table 2, it is observed that the eight items are grouped under two factors with eigenvalues greater than 1. According to Reise et al. [31], factors with eigenvalues greater than 1 can be considered significant. Additionally, based on the Kaiser Criterion (eigenvalue > 1), these two factors together explain 74.628% of the total variance. Moreover, the rotated factor loadings were found to be 3.057 for the first factor and 2.913 for the second factor. The Scree Plot illustrating the factor loadings of the ECS is presented in Figure 1.

When examining Figure 1, the scree plot displays the eigenvalues associated with each component of the scale. The graph indicates a significant drop after the second component, with eigenvalues exceeding 1 for the first two components. In accordance with the Kaiser Criterion, this sharp decline in eigenvalues after the second component suggests that the data are best represented by two factors. The remaining components have eigenvalues below 1, indicating that they contribute less to the explanation of variance. The rotated component matrix is presented in Table 3.

**Table 3:** Rotated component matrix.

Item	Factor 1	Factor 2
1. When I do not exercise, my desire to exercise emerges intensely.	0.867	
2. When I do not exercise, I frequently think about exercising.	0.905	
3. When I do not exercise, I feel a mental obsession with exercising.	0.711	
4. When I do not exercise, I feel restless.	0.773	
5. When I feel the urge to exercise, I have difficulty controlling it.		0.838
6. On days when I cannot exercise, my mood is significantly negatively affected.		0.732
7. When I cannot exercise, my ability to concentrate significantly decreases.		0.769
8. My need to exercise makes it difficult for me to enjoy other activities I find pleasurable.		0.795
9. When I am unable to engage in exercise, my desire to exercise negatively affects my daily activities.	0.488	0.484*

* During the item analysis, item 9 was found to exhibit similar factor loadings on both factors (cross‐loading) and was therefore removed from the final scale structure.

The rotated component matrix demonstrates that the eight items are grouped into two distinct factors. Items 1 through 4, with factor loadings ranging from 0.711 to 0.905, strongly cluster under Factor 1, indicating a shared underlying structure. Similarly, items 5 through 8, with loadings between 0.732 and 0.838, form Factor 2, representing a separate construct (Table 3). The CFA results are presented in Table 4.

**Table 4:** Confirmatory factor analysis results.

Model fit index	First level	Excellent fit criterion	Acceptable fit criterion	References
*χ* ^2^	32.574	0 ≤ *χ* ^2^≤ 2sd	2sd ≤ *χ* ^2^≤ 3sd	
df	14			
*χ* ^2^/df	2.327	0 ≤ *χ* ^2^/sd≤ 2	2 ≤ X^2^/sd≤ 3	Kline (2015)
*p*	0.00	0.05≤ *p* ≤ 1.00	0.01≤*p* ≤ 0.05	Schermelleh–Engel vd. (2003)
RMSEA	0.077	0.00 ≤ RMSEA ≤ 0.05	0.05 ≤ RMSEA ≤ 0.08	Hu and Bentler (1999)
SRMR	0.030	0.00 ≤ SRMR ≤ 0.05	0.05 ≤ SRMR ≤ 0.08	Hu and Bentler (1999)
GFI	0.999	0.95 ≤ GFI ≤ 1.00	0.90 ≤ GFI ≤ 0.95	Kline (2015)
NFI	0.992	0.95 ≤ NFI ≤ 1.00	0.90 ≤ NFI ≤ 0.95	Bentler and Bonett (1980)
TLI	0.990	0.95 ≤ TLI ≤ 1.00	0.90 ≤ TLI ≤ 0.95	Hu and Bentler (1999)
CFI	0.995	0.95 ≤ CFI ≤ 1.00	0.90 ≤ CFI ≤ 0.95	Bentler (1990)
IFI	0.995	0.95 ≤ IFI ≤ 1.00	0.90 ≤ IFI ≤ 0.95	Bollen (1989)

*Note:* Chi‐square (*χ*²/df); comparative fit index (CFI); Tucker–Lewis Index (TLI); Bentler–Bonett normed fit index (NFI); Bollen's incremental fit index (IFI); root mean square error of approximation (RMSEA); standardized root mean square residual (SRMR); goodness of fit index (GFI).

**Figure 2:**
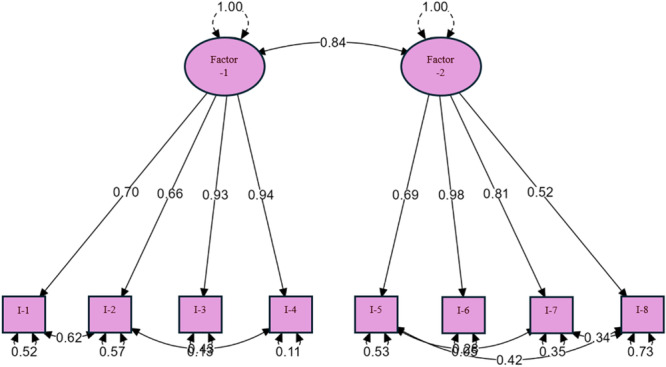
Standardized item loadings from CFA results.

The results of the CFA indicate that the model demonstrates strong overall fit. The Chi‐square to degrees of freedom ratio (*χ*²/df) is 2.327, which falls within the acceptable range as specified by Kline [32]. The *p* value of 0.00 shows that the model is statistically significant. The RMSEA value is 0.077, which is within the acceptable range suggested by Hu and Bentler [33]. Similarly, the SRMR value of 0.030 reflects an excellent fit of the data. The GFI is 0.999, indicating a near‐perfect model fit. Additionally, other fit indices such as NFI, TLI, CFI, and IFI all exceed 0.99, surpassing the excellent fit thresholds recommended by various researchers [33, 34, 35]. These findings demonstrate that the two‐factor structure identified through EFA aligns well with all the model evaluation criteria, showing strong fit to the data. The standardized ıtem loadings from CFA results are presented in Figure 2.

The CFA model shown in Figure 2 illustrates the relationships between two latent factors, Factor‐1 and Factor‐2, and their corresponding observed variables (I‐1 to I‐8). Factor‐1 is associated with variables I‐1 to I‐4, with factor loadings ranging from 0.66 to 0.94, indicating a strong relationship between the latent factor and the observed variables. Similarly, Factor‐2 is linked to variables I‐5 to I‐8, with factor loadings ranging from 0.52 to 0.98, reflecting equally strong associations. The correlation between the two latent factors is 0.84, demonstrating a significant relationship between the structures they represent. The error variances for the variables range between 0.11 and 0.57, indicating the portion of variance in each variable that is not explained by the latent factors. The item analysis results are presented in Table 5.

**Table 5:** Item analysis results.

Factor	Item number	Standardized item loadings	*R*²	Standard error
Factor‐1	1	0.695	0.483	0.024
	2	0.658	0.433	0.034
	3	0.934	0.872	0.021
	4	0.944	0.892	0.021
Factor‐2	5	0.687	0.472	0.027
	6	0.976	0.952	0.023
	7	0.806	0.649	0.024
	8	0.519	0.269	0.030

According to Table 5, for Factor‐1, which includes items 1–4, standardized factor loadings range from 0.658 to 0.944, and squared multiple correlations (*R*²) vary between 0.433 and 0.892, indicating that these items explain a significant portion of the variance. The standard errors for these items are low, ranging from 0.021 to 0.034, signifying high precision in the estimates. Similarly, Factor‐2, composed of items 5–8, has standardized loadings ranging from 0.519 to 0.976, with *R*² values between 0.269 and 0.952. The standard errors for Factor 2 range from 0.023 to 0.030.

### 3.2 Scale Reliability

The reliability and convergent validity of the measurement instrument were assessed using CR, AVE, and the reliability coefficient obtained through the test‐retest method. For Factor 1, the CR value was calculated as 0.888, the AVE value as 0.670, and the Cronbach's alpha coefficient as *α* = 0.887. These findings suggest that the items under Factor 1 exhibit good internal consistency and explain a significant portion of the variance. Similarly, for Factor 2, the CR value was 0.843, the AVE value was 0.585, and the Cronbach's alpha coefficient was found to be *α* = 0.849, confirming sufficient reliability and convergent validity for this factor. Furthermore, the reliability coefficient assessed through the test‐retest method was determined to be *α* = 0.85 for Factor 1 and *α* = 0.81 for Factor 2, further supporting the scale's reliability over time. Experts emphasize that all CR values should be higher than their respective AVE values [36]. Additionally, it is noted that an internal consistency coefficient of 0.70 or higher is required for acceptable reliability [37]. Accordingly, the results obtained in this study meet these criteria, demonstrating the reliability and validity of the scale.

## 4 Discussion

The present study aimed to develop and validate a measurement tool to assess exercise craving among individuals deprived of exercise. The results of the exploratory and confirmatory factor analyses confirmed an eight‐item, two‐factor structure, demonstrating strong psychometric properties. The factors, named exercise obsession and effects of deprivation, capture the cognitive‐emotional preoccupation with exercise and the negative impact of exercise deprivation on mood and daily functioning, respectively. Together, these factors explain a substantial proportion of the variance, and their strong factor loadings provide evidence for the construct validity of the scale.

The reliability analyses, including Cronbach's alpha, (CR), and test‐retest procedures, demonstrated that the scale has high internal consistency and temporal stability. These findings suggest that the ECS provides a reliable and valid instrument for measuring exercise craving, with potential applications in both research and practice. Specifically, the ECS may be useful in identifying individuals at risk of exercise addiction or in understanding the psychological burden of exercise deprivation in different populations.

Despite these promising results, several limitations should be acknowledged. First, the participant group did not exhibit a homogeneous distribution in terms of demographic characteristics, which may affect the generalizability of the findings. Second, excluding individuals who had only recently developed exercise habits limited the ability to examine craving in novice exercisers. Third, the reliance on self‐report measures may have introduced biases, as participants could provide inaccurate or socially desirable responses. Finally, the items of the scale were generated primarily from literature review and expert input from exercise, health, and measurement specialists, which may have restricted the comprehensiveness of the scale content.

Future research should address these limitations by applying the ECS to more diverse and representative samples, including those who are new to exercise. In addition, studies could incorporate objective behavioral or physiological measures alongside self‐reports to strengthen the validity of findings. Expanding the development of the scale with input from a broader range of experts and populations may further enhance its robustness and applicability.

The English version of the scale can be found in Appendix 1, while the Turkish version is provided in Appendix 2. Since the original scale was developed in Turkish, researchers intending to use the English version are advised to conduct construct validity and reliability analyses before implementation.

## 5 Conclusion

This study successfully developed and validated the ECS, an eight‐item, two‐factor instrument with strong psychometric properties. The scale demonstrated high construct validity, internal consistency, and temporal reliability. By capturing both the cognitive‐emotional preoccupation with exercise and the negative consequences of exercise deprivation, the ECS provides a meaningful framework for assessing exercise craving. The ECS holds promise as a practical and reliable tool for researchers and practitioners seeking to evaluate exercise craving and its potential relationship with exercise addiction. Its use in diverse populations and contexts can contribute to a more comprehensive understanding of the psychological aspects of exercise behavior.

## Author Contributions

**İsmail İlbak:** conceptualization, methodology, investigation, writing – original draft, writing – review and editing. **Mehmet Akarsu:** methodology, formal analysis, writing – review and editing. **Ahmet Yasuntimur:** data curation, investigation, and writing – original draft. **Serkan Düz:** investigation, resources, writing – review, and editing. **Gabriela Kavalirova:** supervision, project administration, writing – review and editing, and validation. **Ladislav Cepicka:** supervision, project administration, writing – review and editing, and validation.

## Funding

The authors received no specific funding for this work.

## Ethics Statement

This study was approved by the İnönü University Social and Human Sciences Research and Publication Ethics Committee (date: 13‐11‐2024; session number: 18; approval number: 17).

## Consent

All participants provided informed consent prior to inclusion in the study.

## Conflicts of Interest

The authors declare no conflicts of interest.

## Transparency Statement

The corresponding author, Ladislav Cepicka, affirms that this manuscript is an honest, accurate, and transparent account of the study being reported; that no important aspects of the study have been omitted; and that any discrepancies from the study as planned (and, if relevant, registered) have been explained.

## Data Availability

The data sets generated and/or analyzed during the current study are available from the corresponding author on reasonable request.

## 1

Table A1

**Table A1:** Exercise craving scale.

No.	Item	Strongly disagree	Disagree	Neutral	Agree	Strongly agree
1	When I do not exercise, my desire to exercise emerges intensely.	1	2	3	4	5
2	When I do not exercise, I frequently think about exercising.	1	2	3	4	5
3	When I do not exercise, I feel a mental obsession with exercising.	1	2	3	4	5
4	When I do not exercise, I feel restless.	1	2	3	4	5
5	When I feel the urge to exercise, I have difficulty controlling it.	1	2	3	4	5
6	On days when I cannot exercise, my mood is significantly negatively affected.	1	2	3	4	5
7	When I cannot exercise, my ability to concentrate significantly decreases.	1	2	3	4	5
8	My need to exercise makes it difficult for me to enjoy other activities I find pleasurable.	1	2	3	4	5

*Note:* The scale consists of two subdimensions: exercise obsession and effects of deprivation. The first four items represent the exercise obsession subdimension, while the last four items represent the Effects of Deprivation subdimension. There are no reverse‐coded items in the scale.

## 1

Table A2

**Table A2:** Egzersiz Aşerme Ölçeği.

No.	Madde	Kesinlikle katılmıyorum	Katılmıyorum	Kararsızım	Katılıyorum	Kesinlikle katılıyorum
1	Egzersiz yapmadığım zamanlarda egzersiz yapma isteğim çok şiddetli ortaya çıkar.	1	2	3	4	5
2	Egzersiz yapmadığım zamanlarda sıklıkla egzersiz yapmayı düşünürüm.	1	2	3	4	5
3	Egzersiz yapmadığım zamanlarda egzersiz yapmaya dair zihinsel bir takıntı hissederim.	1	2	3	4	5
4	Egzersiz yapmadığım zamanlarda, kendimi huzursuz hissederim.	1	2	3	4	5
5	Egzersiz yapma isteği hissettiğimde bunu kontrol etmekte zorlanırım.	1	2	3	4	5
6	Egzersiz yapamadığım günlerde, ruh halim belirgin şekilde olumsuz etkilenir.	1	2	3	4	5
7	Egzersiz yapamadığımda, odaklanma yeteneğim belirgin şekilde azalır.	1	2	3	4	5
8	Egzersiz yapma ihtiyacım, diğer keyif aldığım aktivitelerden zevk almamı zorlaştırır.	1	2	3	4	5

*Note:* Ölçek, iki alt boyuttan oluşmaktadır: Egzersiz Takıntısı ve Yoksunluk Etkileri. İlk dört madde Egzersiz Takıntısı alt boyutunu, son dört madde ise Yoksunluk Etkileri alt boyutunu temsil etmektedir. Ölçekte ters kodlanan madde bulunmamaktadır.
